# The Heavy-Ion Beam Diagnostic of the ISTTOK Tokamak—Highlights and Recent Developments

**DOI:** 10.3390/s22114038

**Published:** 2022-05-26

**Authors:** A. Malaquias, I. S. Nedzelskiy, R. Henriques, R. Sharma

**Affiliations:** 1 Instituto de Plasma e Fusão Nuclear, Instituto Superior Técnico, Universidade de Lisboa, Av. Rovisco Pais, 1049-001 Lisboa, Portugal; igorz@ipfn.tecnico.ulisboa.pt (I.S.N.); rhenriques@ipfn.tecnico.ulisboa.pt (R.H.); 2 Culham Science Centre, Culham Centre for Fusion Energy, United Kingdom Atomic Energy Authority, Abingdon OX14 3DB, UK; ridhima.sharma@ukaea.uk

**Keywords:** nuclear fusion diagnostics, heavy-ion beam probe, heavy-ion beam diagnostic, plasma potential measurements, plasma poloidal field measurements

## Abstract

The unique arrangement of the heavy-ion beam diagnostic in ISTTOK enables one to measure the evolution of temperature, density and pressure-like profiles in normal and AC discharges. The fast chopping beam technique provided the possibility to reduce the noise on the measurements of the plasma pressure-like profile and for the precise control of the plasma column position in real time. The consequent improvements in S/N levels allowed the observation of the effects of runaway beam magnetic energy conversion into plasma local heating. In addition, it made it possible to follow the evolution of the quiescent plasma maintained during AC transitions when the plasma current is null. The use of a new operation mode in the cylindrical energy analyzer provided an improved resolution up to five times in determining the fluctuations of the plasma potential as compared to the normal operation mode. Such analyzer is extremely compact (250 mm × 250 mm × 120 mm) and provides a unique geometry in order to cover the whole plasma diameter. The detector configuration choice gives the possibility for the simultaneous measurements of plasma poloidal magnetic field, plasma pressure-like and plasma potential profiles together with their fluctuations.

## 1. Introduction

The operation of magnetic confinement fusion devices requires the use of sophisticated diagnostic techniques in order to infer on the status of the plasma equilibrium and its evolution. The goal is to optimize the plasma pressure (the product of density and temperature) for maximum energy and particle confinement time in a quasi-stable equilibrium. There are many factors that can affect the performance of the fusion plasma such as impurity content, evolution of magnetohydrodynamics (MHD) modes, density and pressure limits, runaway electron (RE) formation, energetic ions loss, turbulence and transport and others. Diagnostic systems that are currently used are based on (i) magnetic and electric sensors, (ii) spectroscopic systems from visible to X-ray wavelengths, (iii) particle or laser-beam-aided diagnostics and (iv) neutron and gamma detection for fusion-rated plasmas. The ideal set of diagnostic data is the collection of plasma parameters from a full 2D poloidal plasma cross section with a few mm of spatial resolution and within tens of microsecond temporal resolution. In addition, magnetic flux surface reconstruction would benefit from 3D measurements based on a toroidal distribution of magnetic sensors (ideally toroidal distributed 2D sets of data). In practice, the experimental data coverage is limited by a combination of diagnostics that can provide measurements from one or a few parameters: (i) a reduced number of local sample volumes, (ii) an integrated tomographic measurement from a collection of lines of sight crossing the plasma into two or more directions (creating a mesh that covers the whole 2D plasma cross section) or (iii) a 1D radial profile or line integrated measurement from a beam- or laser-added diagnostic. This data collection is treated with the support of self-consistent equilibrium and transport codes in order to provide the status of the plasma and its evolution. Most useful in this evaluation process are diagnostics that can measure several plasma parameters from the same location at the same time instant. Moreover, if the measurements could cover the whole 2D plasma section, it would allow for a more precise calibration of MHD and transport codes’ free parameters.

The heavy-ion beam diagnostic installed on the ISTTOK tokamak (Lisbon, Portugal) has been conceptualized in order to provide simultaneously the plasma radial profile evolution of the plasma temperature, electron density, plasma poloidal magnetic field and plasma potential. In fact, this diagnostic has the capability to scan the plasma in 2D, although in ISTTOK, it is limited in its coverage by geometrical factors. It can in practice provide a 1D full-diameter profile of each of the above parameters.

This paper describes the capabilities that have been developed in this diagnostic and includes a more detailed description of the physics basis for the measurement of the plasma poloidal magnetic field profile in ISTTOK.

## 2. The Heavy-Ion Beam Probe Operation Principle (Classic Configuration)

The original proposal for the heavy-ion beam probe (HIBP) was made in the US in 1970 [1]. A review of methods employed in this configuration of the diagnostic is given in [2]. At about the same time, the Russian Federation scientists started to employ the HIBP in several of their fusion devices. An overview of their work can be obtained in [3].

**Figure 1:**
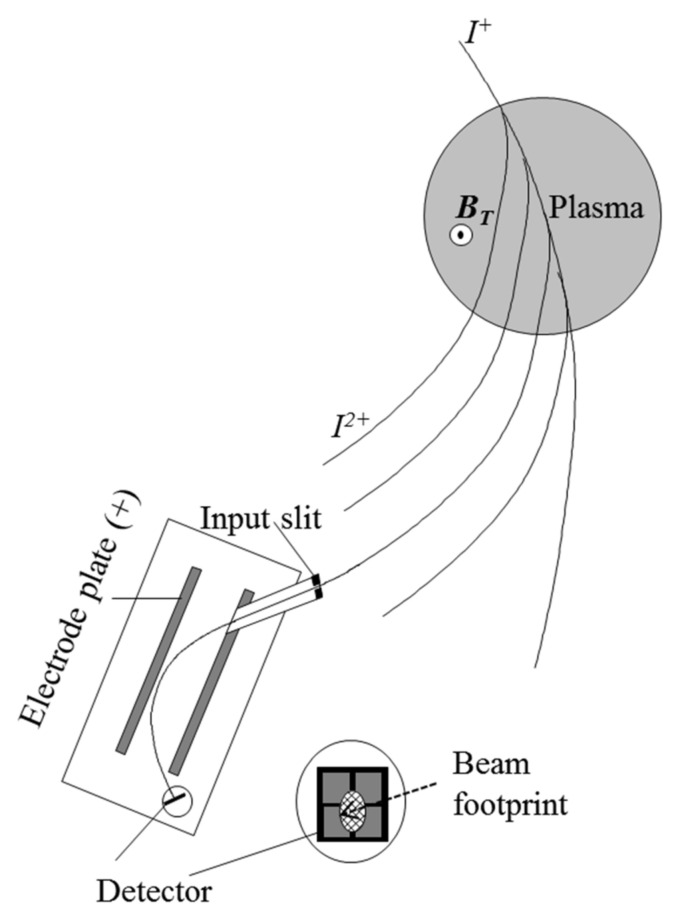
Illustration of the operation of a heavy-ion beam probe (HIBP). A heavy-ion beam *I^+^* is injected into the plasma perpendicularly to the toroidal magnetic field (*B_T_*) producing a quasi-circular trajectory due to the Lorentz force. While colliding with the plasma electrons, the primary beam (*I^+^*) is ionized and produces a fan of secondary beams along the plasma column (*I^2+^*). The trajectory of each secondary beam is determined by half of the Larmor radius with respect to the primary beam.

For the application in a tokamak or stellarator device, a beam of single ionized heavy ions *I^+^* (e.g., *Cs^+^*, *Rb^+^*, etc.) is injected into the plasma (this is called the primary beam) as depicted in Figure 1.

**Figure 2:**
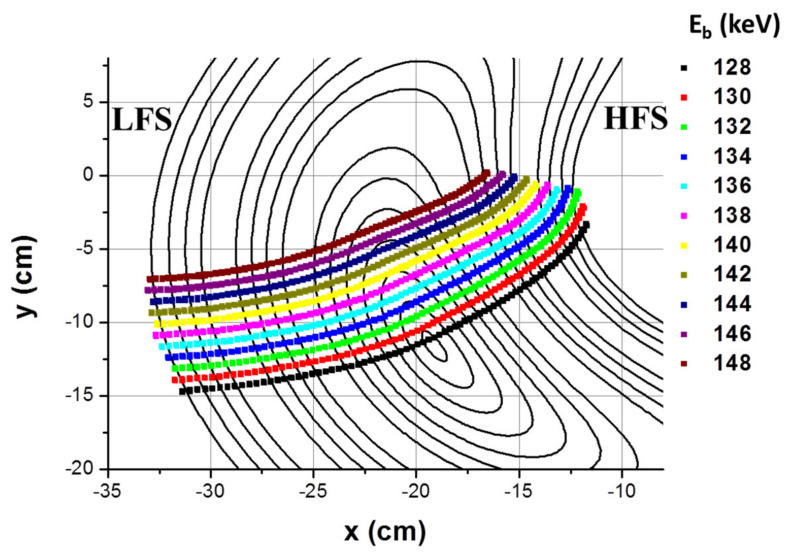
HIBP detector grid for the vertical cross section of TJ-II for *Cs^+^* probing ions. The detector grid consists of 11 detector lines (in different colors) of equal energy E_b_. Each detector line scans the plasma cross section from high-field side to low-field side. (Reprinted from [4], with the permission of AIP Publishing.)

The injection angle is chosen to be perpendicular to the toroidal magnetic field. While crossing the plasma, the primary beam will be further ionized due to collisions with the plasma electrons (and ions if the ion temperature exceeds 2 keV) producing a fan of secondary beams *I^2+^* (e.g., *Cs^2^*^+^, *Rb^2+^*, etc.). The Lorentz force, due to the toroidal magnetic field, imposes a quasi-circular trajectory for the primary beam and for the emerging secondary beams, having half of the Larmor radius of the primary beam. Further down, the detection system collects a secondary beam that is produced over the primary beam elementary path length *dl*. This defines the sample volume dV=dl·A (here *A* is the beam cross-section area) at the position *r* = *ρ* in the plasma. In order to scan the sample volume *dV* over the plasma cross section, the primary beam energy and angle are changed resulting in a grid of energy lines and angles [4]. An example of the scanning energy map is depicted in Figure 2 for the case of the HIBP installed in the TJ-II stellarator.

### 2.1. Plasma Potential Measurements

The most widely used detector in the HIBP configuration is the Proca-Green 30^◦^ parallel plate energy analyzer [5]. As illustrated in Figure 1, it consists of an input slit, a set of parallel conducting plates and an exit aperture followed by a split plate detector. The detector collects the beam intensity and allows one to evaluate its position based on the collected current in each individual cell. The secondary beam energy is obtained by the position of the beam in the detector as it results from the parabolic trajectory inside the electric field produced by the analyzing parallel plates.

The HIBP measures the local plasma potential at the sample volume location via the measurement of the secondary beam energy. These two quantities can be related by solving the energy conservation equation for the primary and secondary beams. The primary beam total energy before entering the plasma at source (*s*) equals the total energy at the ionization point (*p*). Further, the secondary beam total energy at ionization point (*p*) equals the total energy at detection point (*d*). Equating these two conditions, we obtain:

(1) $$\left\{\begin{array}{c} K_{1s}+eV_{s}=K_{1p}+eV_{p}\\ K_{2p}+2eV_{p}=K_{2d}+2eV_{d} \end{array}\right.\;\;$$

Noting that at the ionization point, the kinetic energy of primary and secondary beams can be taken to be equal (as there is negligible momentum transfer from the electron to the primary ion), we can use *K*_1*p*_ *≈ K*_2*p*_ and obtain, by subtraction of the above equations, the potential at the ionization point p,

*V_p_ = (K*_2*d*_*− K*_1*s*_*) + (*2*V_d_ − V_s_)* (2)

Thus, *V_p_* is only a function of the secondary beam energy as the injection energy of the primary beam is known and the potential at the exit of the source and detector is also known, usually kept at ground potential.

### 2.2. Plasma Density Fluctuation Measurements

The secondary beam carries also information about the plasma density on its current intensity. The collected current results from a source contribution and the integral attenuation of primary and secondary beams; it is therefore an integrated measurement. The current of each secondary beam detected is governed by

(3) $$I_{d}^{2+}=2\cdot I_{s}^{+}\cdot n\left(r\right)\cdot \sigma_{12}\left(T\right)\cdot dl\cdot A\cdot B,$$

where the term on the right contains (i) the ratio of charge between primary and secondary beam (*I^2^*^+^/*I^+^* = 2), (ii) the source factor of the secondary beam generation given by the product of the density at the ionization point and the effective cross section of the ionization process ($I^{+}+e\to I^{2+}+2e$), $n\left(r\right).\sigma_{12}\left(T\right)$, at the same location (this is usually called the ‘*n sigma*’ product), and (iii) the path length *dl* over which the ionization takes place (this is determined by the detector cell size projected over the primary beam trajectory). The terms *A* and *B* account respectively for the attenuation suffered by the primary beam from the plasma entry until the ionization volume location and the attenuation of the secondary beam from the ionization volume location until the plasma exit. These terms are explicitly given hereinafter. The effective cross section, $\sigma_{12}\left(T\right)$, is a function of plasma temperature and is calculated from the cross-section data (experimental and/or theoretical) for the reactions of interest. The calculation integrates in the velocity space the reactions’ cross sections over the equilibrium Maxwellian velocity distribution of the plasma electrons. This integration is performed for each value of *T* covering the range of temperatures of interest.

The HIBP in this classical configuration has provided the measurements of local plasma potential and density fluctuations in many devices around the world. For example, the T-10 tokamak HIBP [6] can measure the cross phase of density oscillations, the poloidal phase velocity of turbulence rotation, the poloidal electric field and the radial turbulent particle flux. The use of spectral analysis techniques and cross correlations with magnetic probe measurements allows one to distinguish between various types of turbulence such as broadband, quasi-coherent, tearing and geodesic acoustic modes.

### 2.3. Plasma Poloidal Field Fluctuation Measurements

The trajectories of the primary and secondary beams are perturbed by the poloidal field produced by the plasma current. The Lorentz force imposes a deflection of the primary and secondary beams along the toroidal direction *z* (or *φ*, i.e., the toroidal magnetic field direction). This deflection can be measured by the position of the beam in the detector. In the case of the Proca-Green 30° parallel plate energy analyzer, the deflection is in the perpendicular direction to the deflection caused by the plasma potential measurements making these two measurements naturally independent. The final toroidal position of the secondary beam $z_{d}^{2+}$ is governed by the following equation given in [3] (in cylindrical approximation)

(4) $$\begin{array}{l} z_{d}^{2+}=z_{0}^{+}+\underset{z^{+}}{\underset{︸}{\frac{e}{m}\int_{\rho_{0}}^{\rho_{j}}B_{\rho }(\rho )(t_{j}-\tau_{1}(\rho ))d\rho }}+\underset{z^{2+}}{\underset{︸}{\frac{2e}{m}\int_{\rho_{j}}^{\rho_{d}}B_{\rho }(\rho )(t_{d}-\tau_{2}(\rho ))d\rho }}+\\ +\underset{A}{\underset{︸}{(t_{d}-t_{j})\left(\frac{e}{m}\int_{\rho_{0}}^{\rho_{j}}B_{\rho }(\rho )d\rho +{\dot{z}}_{0}^{+}\right)}} \end{array}$$

The different terms on the right side contributing to this deflection are (i) the initial injection position of the primary beam, $z_{0}^{+}$, (ii) the deflection that the primary beam suffers from the source to the ionization point, $z^{+}$, (iii) the deflection that the secondary beam suffers from the ionization point to the detector, $z^{2+}$, and (iv) the term *A* corresponding to the contribution imposed by the increment in the secondary beam velocity at the ionization volume due to the local poloidal magnetic field times the travel time, plus the velocity in the *z*-direction of the primary beam at the ionization point.

In the particular case where local MHD activity is present at the ionization volume, having some characteristic fluctuation spectra, it is possible to link the detector beam position fluctuations with the oscillations of the poloidal field. This constitutes the basis for MHD fluctuation studies such as, for instance, the Alfvén eigenmode studies presented in [7]. In the most general case, the final position of the beam measured in the detector is integrated over many contributions and might not be uniquely related to the plasma poloidal field strength at the sample volume calling for a more in-depth evaluation of each term contribution.

## 3. The Heavy-Ion Beam Diagnostic Operation Principle

The path integral limitations mentioned above can be accounted for if a different configuration is used for the diagnostic. In order to distinguish it from the classical configuration, it is denominated the heavy-ion beam diagnostic (by replacing the word ‘probe’ with ‘diagnostic’). It is based on a multiple-cell array detector (MCAD) that collects all the fan of secondary beams at the same time. This approach avoids the need to scan the primary beam in order to obtain 1D full-diameter profiles as they are provided quasi-instantaneously. It allows, in this way, preserving the phase information between the plasma fluctuations (up to the time limit that the beam takes to travel the plasma column: circa 0.5 microseconds in ISTTOK).

**Figure 3:**
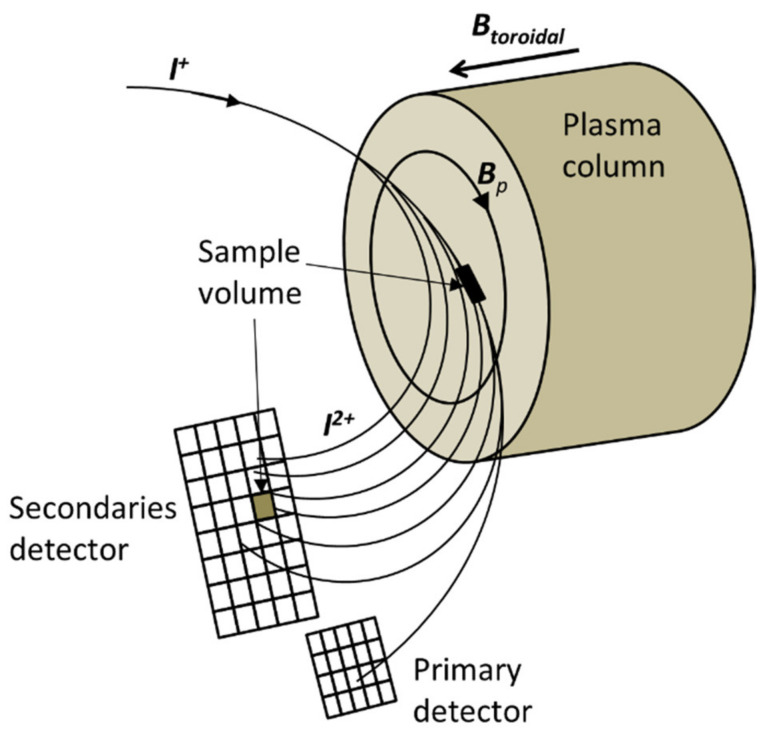
Schematic configuration of the heavy-ion beam diagnostic (HIBD). In this arrangement, all the secondary ions and the primary beam are detected in a multiple-cell array detector (MCAD).

The operation of the HIB diagnostic is illustrated in Figure 3. The main difference from the HIB-probe arrangement is that all secondary and primary beams are ‘instantaneously’ collected in a multiple-cell array detector (MCAD). The beam finite width (in the toroidal direction) will be captured by the cells in the same row. Adding all the currents in one row provides the total current collected from the sample volume *dl·A*, while the current distribution on the individual cells in one row allows the determination of the toroidal position of the beam.

**Figure 4:**
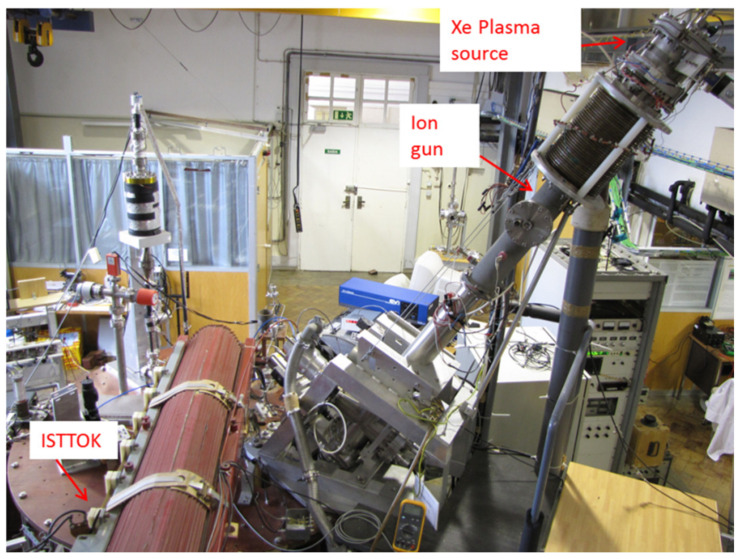
Set-up of the HIBD in ISTTOK tokamak.

In ISTTOK, this configuration was adopted. The ion gun injects a 3 micro-ampere, 20–25 keV energy beam of *Xe^+^* (or *Cs^+^, Hg^+^*) into the ISTTOK plasma. ISTTOK plasma has an 8.5 cm minor radius and 4–6 kA of plasma current confined in a 0.48 T toroidal magnetic field (Figure 4).

In the following sections are presented the algorithms developed to account for the path integral effects and recover the local information along the primary beam path. Examples of experimental data retrieved by these methods are also presented.

### 3.1. The Determination of Local Values of the ‘n sigma’ Profile

**Figure 5:**
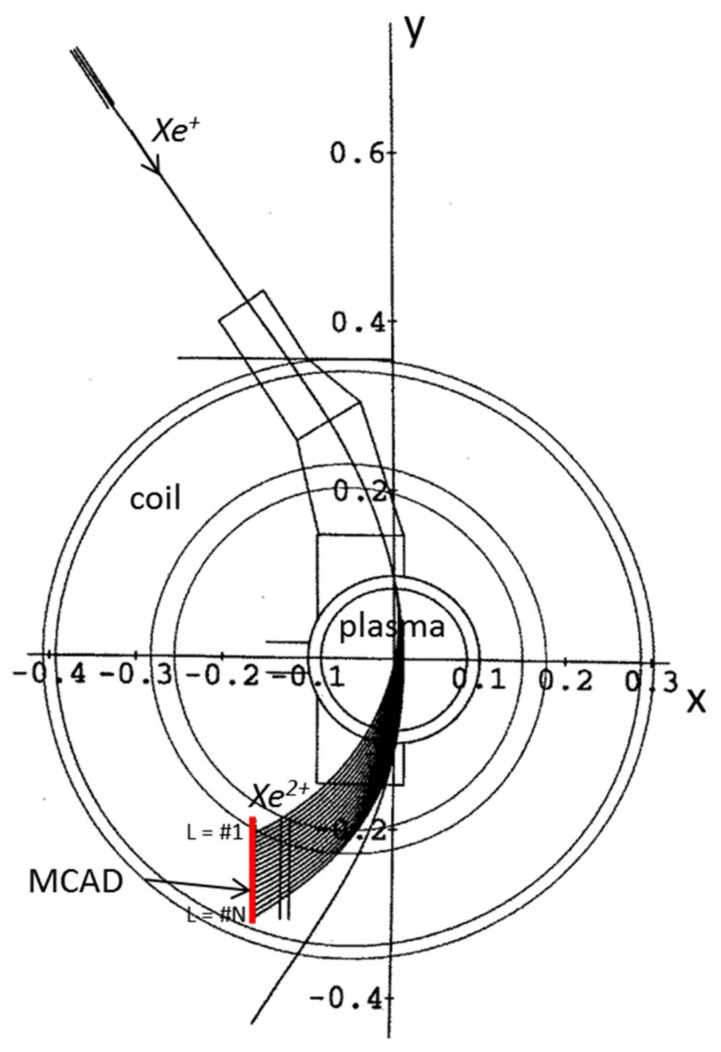
Trajectories (units in *m*) of a 22 keV primary *Xe+* beam and corresponding cloud of secondary beams as calculated by the HIBD modeling code for the tokamak ISTTOK (*B_T_* = 0.48 T). The detector, represented by the red line, has N rows in total, each indexed by the number L (#1, …, #N) (reprinted from [8]).

The development of a retrieving method for the local values of ‘*n sigma*’ (here also referred to as ‘pressure-like’ due to the dependence of effective cross section σ_12_, on the plasma temperature) has been detailed in [8]. The methodology is mainly based on the differentiation (starting from the first row of detector cells) of the currents collected on neighboring rows, being the difference related to the attenuation current of the primary beam between the two cells. In Figure 5, is depicted a simulation of the trajectories in ISTTOK (for a *Xe^+^* beam, 22 keV) and the chosen detector line arrangement.

The attenuation processes that are relevant for ISTTOK comprise the electron impact ionization reactions of (i) *I^+^ + e* → *I*^2*+*^ *+* 2*e*, (ii) *I^+^ + e* → *I*^3*+*^ *+* 3*e* and (iii) *I*^2*+*^ *+ e* → *I*^3*+*^ *+* 2*e*. The complete equation describing the contribution of these processes in the generation of the detected currents is (where the terms ***A*** and ***B*** of Equation (3) are here explicitly given)

(5) $$\begin{array}{l} I_{j(\det)}^{2+}=2I_{0}^{+}\underset{A}{\underset{︸}{\exp\left(-\int_{R_{i}}^{r_{j}}n_{e}(s_{1})\left({\hat{\sigma }}_{12}+{\hat{\sigma }}_{13}\right)ds_{1}\right)\times }}\\ n_{e}(r_{j}){\hat{\sigma }}_{12}(r_{j})dl_{\left(cell\right)}\underset{B}{\underset{︸}{\exp\left(-\int_{r_{j}}^{Rp}n_{e}(s_{2}){\hat{\sigma }}_{23}ds_{2}\right)}} \end{array}$$

As was demonstrated in [8], the determination of the $n{\hat{\sigma }}_{12}(r_{j})$ relative profile can be performed by subtracting the primary beam current entering the ionization volume from the value of the detected secondary beam current obtained in the previous ionization volume (weight by the charge ratio between *I^+^* and *I*^2*+*^). Therefore, the primary current available at each ionization volume (*j* = 1, …, N) can be obtained from

(6) $$I_{j}^{+}=I_{0}^{+}-\sum_{L=0}^{j-1}\frac{1}{2}I_{L(\det)}^{2+}$$

with the definition that for *L =* 0, there is no current (no cell for *L =* 0). Making the ratio between the secondary current and the primary current corresponding to the same sampling volume, for each ionization volume *j*, we can write for the generation factor (ignoring, for now, the terms *A* and *B* in Equation (3))

(7) $$n_{e}{\hat{\sigma }}_{12}\left(r_{j}\right)=\frac{I_{j\left(det\right)}^{2+}}{2I_{j}^{+}dl}$$

obtaining in this way its relative profile along the primary beam path (by scanning for all values of *j*). The absolute profile of ‘*n sigma*’ can be computed by using the relative ‘*n sigma*’ profile combined with the information about the total current lost by all secondary beams traveling in the plasma. This current is given by the difference between the primary beam injected current minus the primary beam collected current, measured after crossing the whole plasma (with the factor 2 accounting for charge conversion) and the total current of the secondaries collected at the detector

(8) $$I_{total.lost}^{2+\to 3+}=2\left(I_{0}^{+}-I_{det}^{+}\right)-I_{det.\;total}^{2+}$$

The current $I_{total.lost}^{2+\to 3+}$ is distributed per each secondary beam weighted by the corresponding ‘*n sigma*’ integral. Each individual integral is taken along the secondary beam path from the ionization volume location until the plasma exit. The computation is performed using the following function

(9) $${ℵ}_{j}^{2\to 3}=\frac{I_{j}^{2+\to 3+}}{I_{total}^{2+\to 3+}}=\frac{\left\{I_{j}^{2+}\int_{r_{j}}^{R_{p}}n_{e}{\hat{\sigma }}_{23}dl\right\}}{\sum_{j=1}^{N}\left\{I_{j}^{2+}\int_{r_{j}}^{R_{p}}n_{e}{\hat{\sigma }}_{23}dl\right\}}$$

The ratio in the above equation can be multiplied by the term $I_{total.lost}^{2+\to 3+}$ allowing one to determine the current that was lost by each secondary beam (here we are using the approximation that the relative profile of $n{\hat{\sigma }}_{12}(r_{j})$ and $n{\hat{\sigma }}_{23}(r_{j})$ are identical, which is confirmed by numerical calculation). This information allows reconstructing the absolute values of $n{\hat{\sigma }}_{12}(r_{j})$ at each ionization volume (to follow more details, including the contribution of the reaction *I^+^ + e* → *I*^3*+*^ *+* 3*e*, please consult [8]).

**Figure 6:**
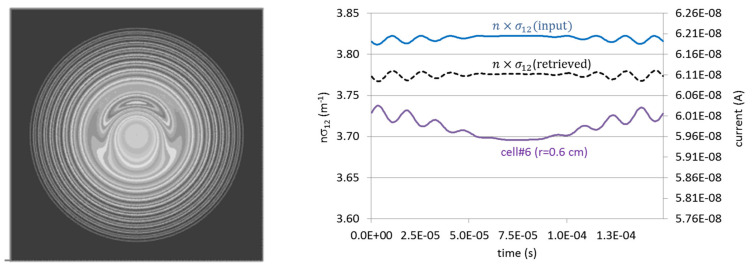
Left: illustration of the ‘*n sigma*’ contour plot of the *m* = 1 mode (plasma radius = 8.5 cm); Right: the evolution of the inputted and retrieved ‘*n sigma*’ (left scale) at the ionization volume in position *r* = 0.6 cm and the evolution of the detector current (right scale) for the secondary ions emerging from the ionization volume (reprinted from [8]).

The importance of obtaining local measurements is not only beneficial for obtaining the absolute profiles but also manifests in improving the detection and characterization of fluctuations (in frequency and phase). To illustrate this, we modeled an equivalent perturbation in the plasma density and temperature induced by an MHD mode number *m* = 1 located at *r* = 0.025 m. The corresponding perturbation on the ‘*n sigma*’ value was ±30% of the local non-perturbed *n sigma* value. Elsewhere, the plasma presented a parabolic density profile and a peak temperature profile. The mode rotation frequency is 67 kHz with a sinusoidal imposed amplitude modulation rate of 6.7 kHz [8]. In Figure 6 left, is depicted the contour plot of ‘*n sigma*’ as inputted in the simulation code; on the right is presented the evolution of the inputted and retrieved $n{\hat{\sigma }}_{12}(r_{j})$ values at a particular location of the central ionization volume (*r* = 0.6 cm) during one amplitude modulation cycle (period T = 1/6700 s). We can observe that the absolute value of ‘*n sigma*’ is retrieved within <2% absolute error. Moreover, the evolution in time of both ‘*n sigma*’ signals inputted and retrieved is in phase. On the same graph, we can see the evolution of the collected secondary current emerging from the ionization volume. The evolution of the collected secondary current preserves information about the frequency of the mode but shows a phase shift between the ‘*n sigma*’ rotation signature of ≈ π (peak and valley relative position offset). This happens because the secondary current in this cell is influenced by the fluctuation of the primary beam current when it passes over the rotating *m* = 1 mode and propagates to the next secondary beam ionization volume.

Depending on the coexistence of other modes and size of their fluctuations, the contamination of the detector signals by the path integral effects of the primary beam can lead to ‘ghost’ coherence and spectral signatures if these integral effects are not removed in the signal analysis. It is important to point out that for the range of average densities in ISTTOK, below 5 × 10^18^ part/m^3^, and due to the relatively small plasma cross section, these effects are not detectable. For higher density plasmas in larger devices, these effects are expected to show a detectable contribution.

#### 3.1.1. Experimental Measurements of Pressure-like Profiles

Experiments have been conducted in ISTTOK [9] where the ‘*n sigma*’ relative profile was measured during MHD activity. Combining the HIBD with the Mirnov coil data, it is possible to identify the mode number (Mirnov) and its location (HIBD).

**Figure 7:**
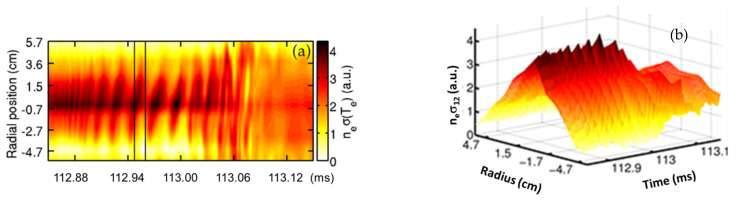
The contour plot of the ‘*n sigma*’ (**a**) and the evolution of the relative profile (**b**) during MHD activity during MHD mode growth leading to the collapse of the plasma pressure profile after *t* > 113.06 ms (reprinted from [9], with the permission of AIP Publishing).

In Figure 7, is depicted the evolution of an MHD instability measured by the HIBD.

From the Mirnov coil signals, this has been identified as a *m* = 2 poloidal number mode. From the HIBD data, the evolution of the mode can be followed and the frequency of the mode rotation can be determined to be circa 63 kHz. This mode grows in size and ends with a disruption (*t* >113.06 ms) leading to a flatness of the ‘*n sigma*’ profile.

**Figure 8:**
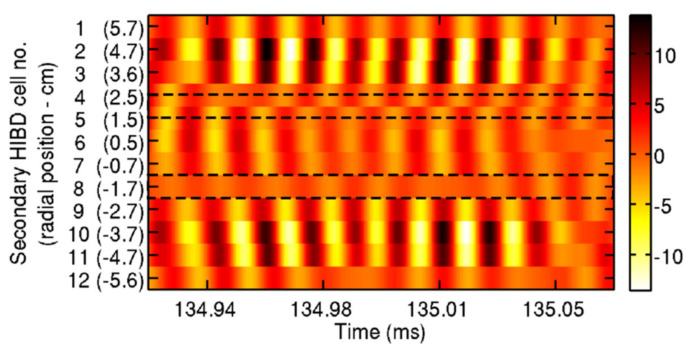
HIBD signals filtered with a 20 kHz bandwidth filter centered at 65 kHz. Dashed line rectangles indicate the location of the phase reversal. The scale is in a.u. (reprinted from [9], with the permission of AIP Publishing).

The relatively highly detailed information that can be retrieved with the HIBD can be used to visualize the radial structure of a tearing mode (TM). For instance, it is expected that in a TM, the inner flux and the outer flux surfaces would exhibit the characteristic oscillation frequency of the mode but with an opposite phase. In Figure 8, the HIBD signal is presented filtered with a central frequency of 65 kHz and a bandwidth of 20 kHz. Using the Mirnov coils, this mode is identified as *m* = 4, and, from the HIBD data, it is found to be located slightly off-center at around *r* = 2 cm. The bars indicate the location of the phase inversion as it is seen directly in the contour plot of the cell currents (peaks and valleys are offset by π rad).

#### 3.1.2. Control of Plasma Column Position

The ‘*n sigma*’ profile can in principle be used to determine the plasma column position along the path of the primary beam by mapping the position in the plasma corresponding to the ‘*n sigma*’ highest value. As the ‘*n sigma*’ is a pressure-like measurement, it shall follow a function closely related to the plasma pressure (this is particularly true for the ISTTOK range of plasma density and temperature) and reflect the position of the plasma column center. In the case of monotonic peaked profiles, the peak value of ‘*n sigma*’ could be used to follow the plasma column center movement (vertically, in ISTTOK). However, in the case of hollow pressure profiles and in the presence of relatively large pressure fluctuations, the first momentum of the ‘*n sigma*’ distribution offers a more robust signal to follow. The determination of the first momentum of the ‘*n sigma*’ function is simply made from the individual detector row positions weighted by the ratio of the local ‘*n sigma*’ to the ‘*n sigma*’ integral value (calculated from the contribution of all detector cells). This will give the centroid of ‘*n sigma*’ that is then mapped from the detector into the plasma radius. This method was used to control the vertical (y) position of the plasma column of ISTTOK in real time [10].

**Figure 9:**
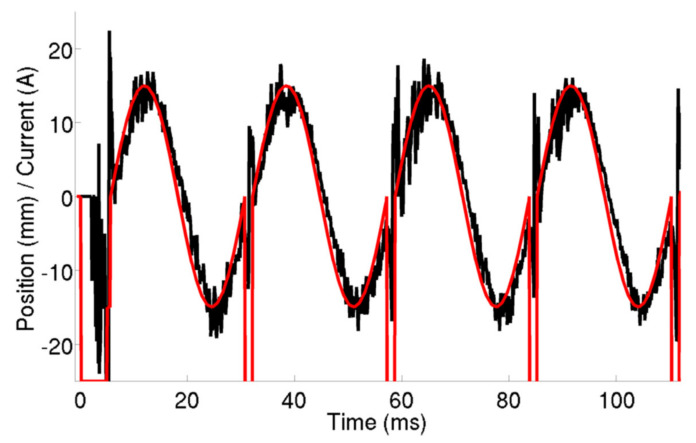
Control of the vertical plasma column position (black line) during an AC discharge by following closely the controller pre-set function (red line). The real-time feedback signal is obtained using the HIBD for retrieving the ‘*n sigma*’ profile. The update frequency of the plasma column vertical position is f = 10 kHz (reprinted from [10]).

In Figure 9 is depicted the evolution of the plasma column vertical position measured by the HIBD during AC operation. The red line corresponds to the pre-set point function given to the vertical position controller. The signals from the HIBD are processed in real time to provide the first momentum of the ‘*n sigma*’ profile and feed it to the vertical controller. The controller compares the experimental results of plasma column position with the pre-set point function and acts in the plasma column (via vertical field coils) to correct the plasma position within a pre-defined interval of acceptance.

#### 3.1.3. Pressure-like Profiles Measured during ISTTOK AC Operation

**Figure 10:**
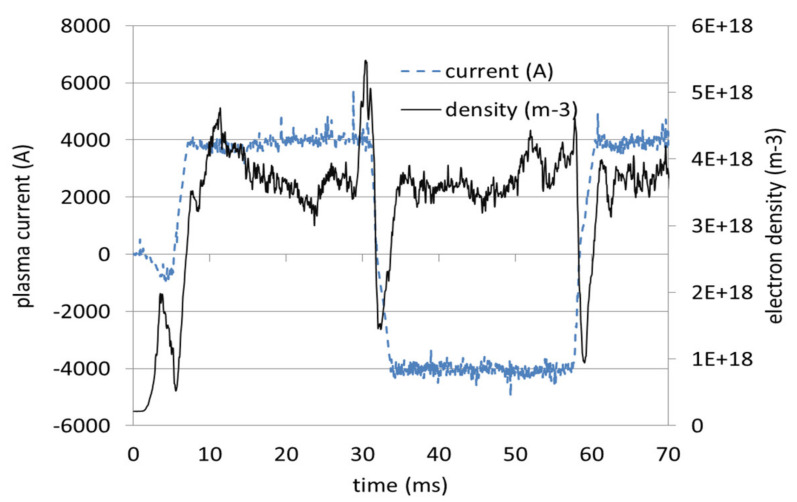
Evolution of plasma current (dashed line) and electron density during one AC cycle.

The ISTTOK tokamak is able to perform alternating discharges up to 1 s. duration with a full AC cycle lasting about 50 ms [11]. The AC transition is characterized by the instant when the total plasma current is null. It is observed that during this instant, the plasma density is not null and presents a local minimum which appears several hundreds of microseconds after the transition (Figure 10).

**Figure 11:**
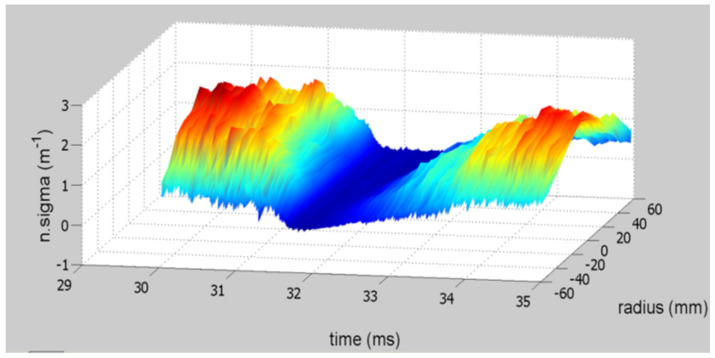
Evolution of the absolute value of ‘*n sigma*’ profile during an AC transition from positive to negative plasma current.

The HIBD has been used to study the plasma pressure-like profile evolution during the AC transition under edge electrode biasing [12]. In these experiments, the beam was chopped in the range of 180 kHz. The instants with the beam off were used to measure the noise in the detector cells and discount it during the beam-on phase (by interpolation between two off cycles). This technique allowed obtaining measurements from relatively low plasma pressures and revealing the evolution of the ‘pressure-like’ profiles during the AC transition. In Figure 11, is depicted the corresponding pressure-like profile evolution during the transition shown above, from positive to negative plasma current.

**Figure 12:**
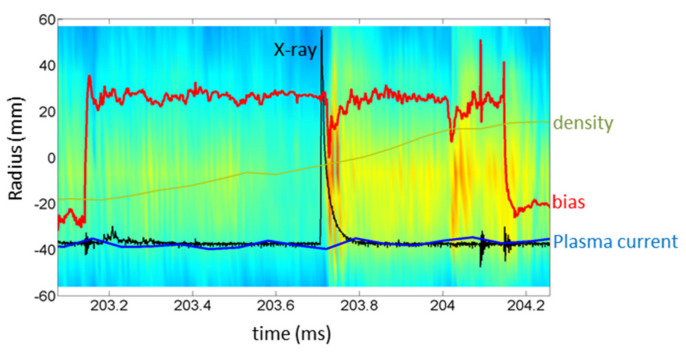
The colored background represents the contour plot of ‘*n sigma*’ evolution (a.u.) covering the plasma radius from about ±50 mm. Superimposed signals show the plasma current, plasma density and electrode biasing voltage (a.u.). It is clearly seen that, following the X-ray signal, the ‘*n sigma*’ profile increases in the plasma core region.

During electrode-biasing-assisted AC operation, it was found that a noticeable amount of runaway electrons can be produced. These runaway electrons can form beams that collide with the limiter or the vessel. In this process, they can input current to the plasma, when converting the loss of magnetic energy associated with its own current. This effect could in principle be detected by the HIBD as a momentary increase in the plasma ‘pressure-like’ signal (due to the plasma temperature increase via Ohm heating). Figure 12 shows the clear increase in the ‘*n sigma*’ (2D contour plot) value in the plasma core just after the X-ray burst (t ≈ 203.5 ms). This observation is due to the conversion of the runaway beam magnetic energy into a plasma pressure increase. Further down in time (*t* > 204 ms) a second burst seems to have occurred as it displays the same kind of signature in the *‘n sigma’* profile although has no X-ray signature associated, probably because the generated X-rays are out of the collection cone of the Si-based detector.

### 3.2. Determination of Plasma Density and Temperature Profiles

**Figure 13:**
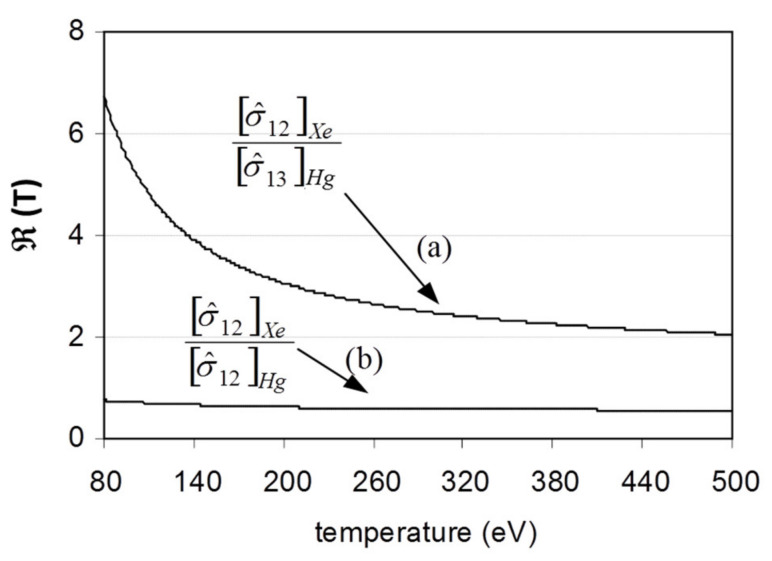
The effective cross-section ratio function for two ionizing reactions.

The retrieving of ‘*n sigma*’ absolute values is most relevant for using the HIBD in temperature and density measurements, i.e., for obtaining the two profiles separately. One method used in ISTTOK [13] is based on the injection of two different beam species. Due to the type of ion source used, it is possible to produce individual or mixed beams of *Cs^+^*, *Xe^+^* and *Hg^+^*. If two different beam species are injected through the same path in the plasma and the corresponding ‘*n sigma*’ absolute profiles are retrieved, then their ratio is only a function of the corresponding ionization effective cross-section ratio. Since the density term is common for both species, we can write

(10) $$ℜ(T_{e})=\frac{{\left[n_{e}\times {\hat{\sigma }}_{1m}\right]}_{I_{1}^{+}}}{{\left[n_{e}\times {\hat{\sigma }}_{1m}\right]}_{I_{2}^{+}}}=\frac{{\left[{\hat{\sigma }}_{1m}(T_{e})\right]}_{I_{1}^{+}}}{{\left[{\hat{\sigma }}_{1m}(T_{e})\right]}_{I_{2}^{+}}}$$

where *m* denotes the charge of the secondary beam. The use of this method calls for the largest possible ratio dependence between the effective cross sections involved such that the function °(T) is strongly dependent on the plasma temperature. For the case of ISTTOK, the chosen reactions and species were the ones that produced the following ions: *Xe ^+^* → *Xe*^2*+*^ and *Hg^+^* → *Hg*^3*+*^. The °(T) function for these reactions is depicted in Figure 13 together with the ratio function for the processes *Xe^+^* → *Xe*^2*+*^ and *Hg^+^* → *Hg*^2*+*^, useful to estimate the current of the *Hg*^2*+*^ ions (which is not collected at the detector) from the collected *Xe*^2*+*^ ions. 

**Figure 14:**
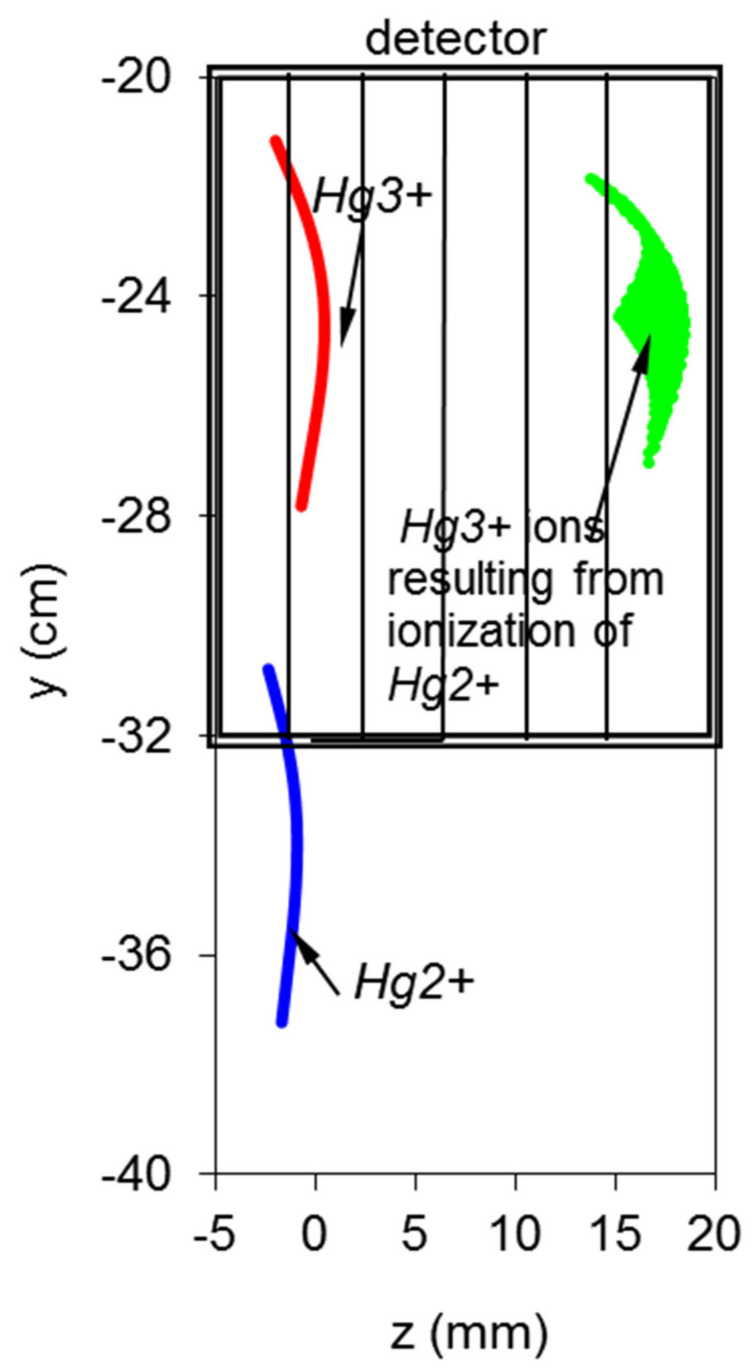
Footprint of *Hg* ions in the detector area. The boxed area corresponds to the MCAD real size.

The trajectory of the ions of interest is evaluated by the simulation code. As depicted in Figure 14, the *Hg*^3*+*^ ions can be spatially discriminated from the *Hg*^2*+*^ ions (which are not detected). The *Xe*^2*+*^ ions are also spatially discriminated from the *Xe*^3*+*^ ions.

**Figure 15:**
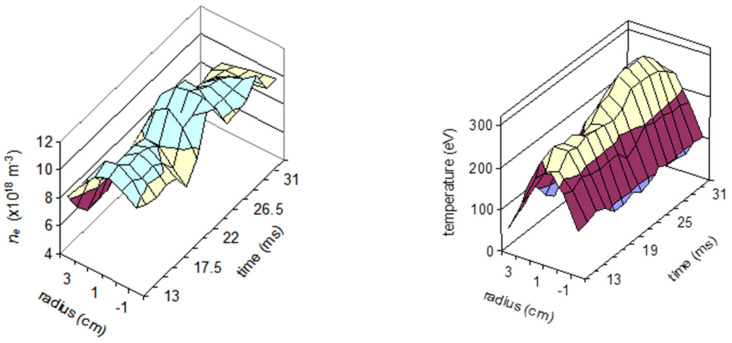
Density (**left**) and temperature (**right**) profiles obtained in ISTTOK with the HIBD using two species injection.

We may note that the reaction *Hg^+^* → *Hg*^3*+*^ only produces measurable signals (in ISTTOK) when the plasma temperature is above 80 eV. This condition constrains the determination of temperature to a typical plasma radius of r ≈ 3 cm. By means of this mixed beam technique, the profiles of density and temperature were measured (Figure 15).

### 3.3. Poloidal Magnetic Field Retrieval and Measurements

The poloidal magnetic field *B_p_*, produced by the plasma current, obeys the Maxwell Equation (Ampere’s Law),

(11) $$\mu_{o}\vec{j}=\vec{\nabla }\times {\vec{B}}_{p}$$

where *j* is the plasma current density. As mentioned above, some works using the HIB-probe configuration reported on the determination of local fluctuations of *B_p_*. The measurement contains the integral effects of primary and secondary beam trajectories and cannot be considered absolutely local. However, in some special conditions, if the local magnetic field fluctuations at the sample volume exceed the path integral effects, then the frequency content of such fluctuations can be manifested in the beam position fluctuations. These are special conditions that require detailed analysis of the data in order to infer on its validity and on the non-contamination by other non-local sources.

On the other hand, the HIB-diagnostic configuration allows for the determination of the local poloidal magnetic field *B_p_*, at each sample volume, by allowing the removal of the path integral effects. The total field at each sample volume will be the sum of the external coils’ fields and the plasma current magnetic field. The external fields can be accounted by calculation or by specific measurements used for establishing the distribution and values of the machine vacuum fields. These known vacuum fields can be subtracted from the total measured field in order to obtain the plasma current poloidal field contribution.

**Figure 16:**
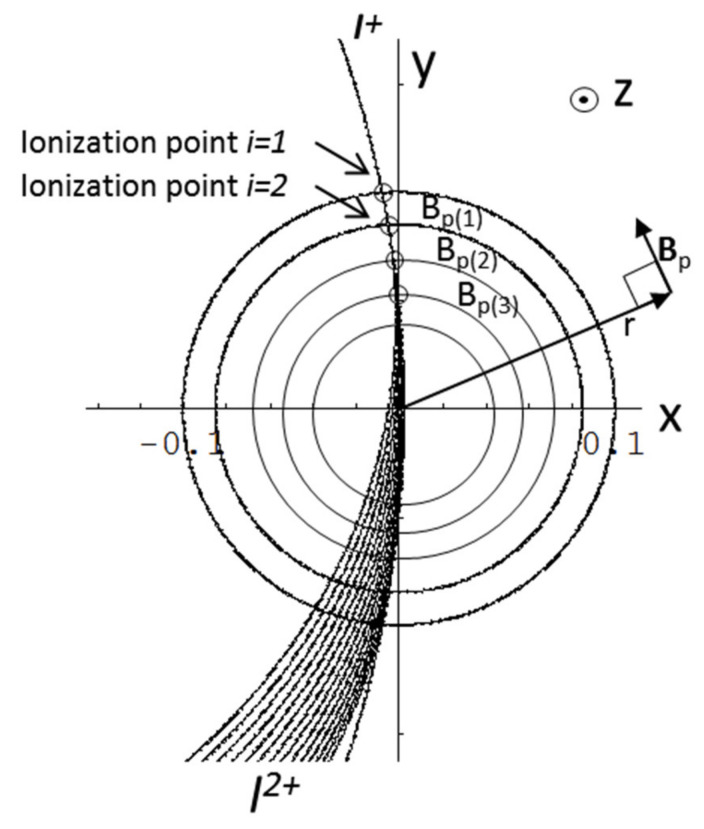
Ion trajectories assuming cylindrical geometry for the plasma current.

Let us start by assuming cylindrical symmetry (which is a good approximation for ISTTOK) and represent the poloidal field flux lines as is depicted in Figure 16. We also assume that the beam trajectories are perpendicular to the flux surfaces (otherwise, the perpendicular component of the poloidal field to the beam trajectory is the one that is considered).

The integral form of Equation (11) can be given inside and outside the plasma column at point *r* as:

(12) $$B_{p}=\frac{\mu_{o}I(r)}{2\pi r}$$

where *I(r)* represents the current enclosed in the cylindrical volume of radius *r*. The values of *I(r)* inside the plasma are dependent on the plasma current density *j(r)* profile by the relation (in cylindrical coordinates)

(13) $$I\left(r\right)=\int_{0}^{r}j\left(r’\right)2\pi rdr’$$

Outside the plasma column, the value of *I(r)* becomes constant and equal to the total plasma current *I_plasma_*.

Once a current profile is defined, the profile of the poloidal magnetic field as a function of plasma radius *r* is given by the combination of Equations (12) and (13). The force exerted by the local poloidal field at each ionization volume imposes a local acceleration of the primary beam (and secondary) in the z-direction (the toroidal direction). If we manage to measure the primary beam local acceleration component that is perpendicular to the poloidal field (i.e., in the *z* or toroidal direction), then the corresponding local poloidal field can be obtained from:

(14) $$\left|B_{p}\right|=\frac{m}{e}\left|\frac{dv}{dt}\right|\frac{1}{v_{⊥}}$$

where *dv/dt* stands for the acceleration in the direction *z* which is perpendicular to both the field lines of ***B****_p_*(r) (see Figure 16) and the primary beam velocity *v*_⊥_ (which is here defined as the velocity component lying in the plane XOY and perpendicular to ***B****_p_*(r) field lines). In ISTTOK, the geometry is such that for the assumed conditions, the primary beam velocity projection in the plane XOY is quasi-perpendicular to the poloidal magnetic field lines along all primary and secondary beam trajectories. This means that the acceleration *z* of the primary is induced by the total plasma current poloidal field at the ionization volume. In the case the angle between the primary beam velocity ***v***_XOY_ and the field lines of ***B****_p_*(r) is not perpendicular, then the beam would feel a local *z-*acceleration weighted by the sine of the angle between these two vectors:

(15) $$\left|\frac{dv}{dt}\right|=|\frac{e}{m}{\vec{B}}_{p}\times {\vec{v}}_{XOY}|=\frac{e}{m}|B_{p}|\cdot |v_{XOY}|\cdot \sin\left(\hat{{\vec{B}}_{p}{\vec{v}}_{XOY}}\right)$$

#### 3.3.1. Retrieving of Local Values of Magnetic Poloidal Field

Considering the geometry in Figure 16, we may write for the secondary beam *z-*position in the detector, for ionization point *i* = 1,

(16) $$z_{d(1)}^{2+}=z_{1}^{+}+{\bar{z}}_{1}^{2+}+v_{1}^{+}T_{2(1)}+\underset{s_{1}}{\underset{︸}{\frac{1}{2}{\bar{a}}_{\left(1\right)}^{2+}T_{p(1)}^{2}}}$$

where $z_{1}^{+}$ is the *z*-coordinate of the primary beam at the ionization point (it is known from the injection position of the source and the computation of the beam trajectory under the known magnetic fields, until the entrance in the plasma), and ${\bar{z}}_{1}^{2+}$ is the increment in the *z*-coordinate of the secondary ion from the plasma edge up to the detector (this term contribution can be accounted by using the total plasma current in Equation (12) as the poloidal field in this region is known). The term $v_{1}^{+}$*T*_2*(*1*)*_ accounts for the increment in the *z*-coordinate of the secondary ion due to the fact that it leaves the primary beam with finite *z*-velocity. At the ionization point, we can write $v_{1}^{+}=v_{1}^{2+}$, and therefore its *z*-coordinate suffers an increment from the ionization point until the detector during its time of flight *T*_2*(*1*).*_ The value of $v_{1}^{+}$ is obtained at the point the primary beam enters the plasma. It is computed by the simulation code and only depends on the total plasma current (as it is determined by the beam trajectory before it enters the plasma). The last term accounts for the *z*-increment due to the plasma integral poloidal field between the ionization point and exit of the plasma. It is written on the basis of an average acceleration on the secondary beam ${\bar{a}}_{\left(1\right)}^{2+}$, which accounts for the integral of the acceleration over the secondary beam trajectory from the ionization point until its exit of the plasma during the traveling time $T_{p(1)}$. This average acceleration (which is unknown) can be related to the corresponding average *z*-velocity of the secondary beam ${\bar{v}}_{\left(1\right)}^{2+}$ using

(17) $${\bar{a}}_{\left(1\right)}^{2+}=\frac{{\bar{v}}_{\left(1\right)}^{2+}}{T_{p(1)}}$$

Combining Equations (16) and (17), we can obtain the average value of the secondary ion velocity counted from the ionization point 1 until the exit of the plasma,

(18) $${\bar{v}}_{\left(1\right)}^{2+}=2\frac{z_{d(1)}^{2+}-z_{1}^{+}-{\bar{z}}_{1}^{2+}-v_{1}^{+}T_{2(1)}}{T_{p(1)}^{}}$$

In fact, this first term can be calculated independently of the plasma current profile. It is given by the difference in the *z-*position of the first secondary beam at the ionization point (which can be taken equal to the primary beam *z*-position at the same point) and the *z*-position of the first secondary beam when it leaves the plasma, divided by the corresponding time of flight. This last position is obtained by taking the beam *z*-position on the detector after discounting the increment induced by the total plasma current from the exit point from the plasma until the detector. This is done using the total plasma current and the 3D simulation code. The flight times of the ions from the ionization point until the detector including the time spent between the plasma exit and the detector are given by the geometric installation of the HIBD in ISTTOK. They also can be obtained using the trajectory simulation code; in the present approximation, the flight times are considered not to be significantly affected by the poloidal field profile or by the total plasma current value.

Using this initialization result, let us consider now the motion equation of the secondary beam emerging from ionization point *i* = 2. We can write a similar relation for the *z*-position in the detector,

(19) $$z_{d(2)}^{2+}={\bar{z}}_{\left(2\right)}^{2+}+z_{\left(2\right)}^{+}+v_{\left(2\right)}^{+}T_{2(2)}+\underset{s_{2}}{\underset{︸}{\frac{1}{2}{\bar{a}}_{\left(2\right)}^{2+}T_{p(2)}^{2}}}$$

where ${\bar{a}}_{\left(2\right)}^{2+}$ represents the average z-acceleration of the secondary beam from ionization point 2 to the detector. In a similar way, the integral acceleration can be given by

(20) $${\bar{a}}_{\left(2\right)}^{2+}=\frac{{\bar{v}}_{\left(2\right)}^{2+}}{T_{p(2)}}$$

**Figure 17:**
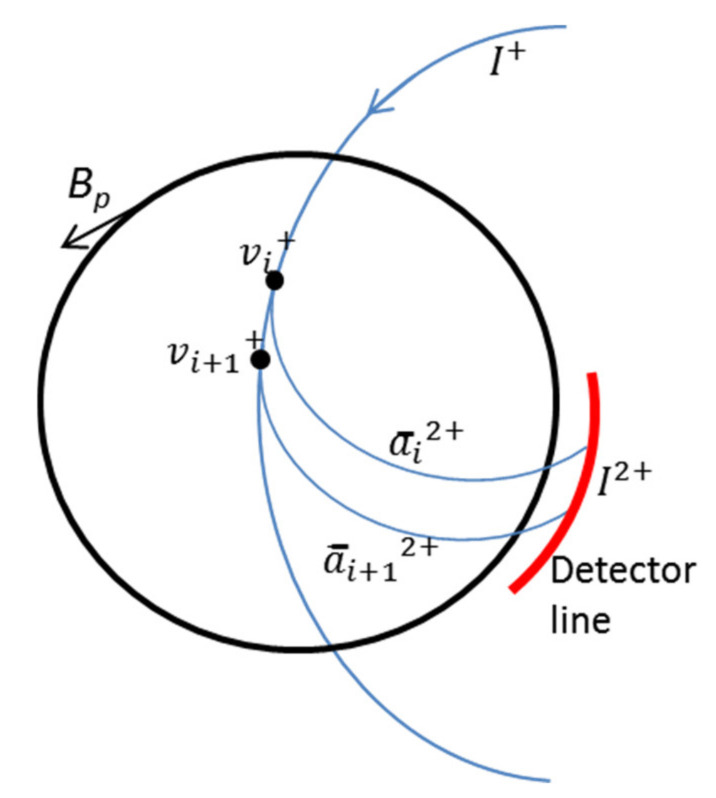
Trajectories of two consecutive secondary beams generated at two very close ionization points.

In Figure 17 is represented the above situation but for the general case of two secondary beams generated at two consecutive ionization points.

For simplicity, we assume that the detector line is just at the plasma exit, and therefore we can ignore in the above equations the z-position increment that each beam suffers from the plasma exit until the detector, ${\bar{z}}_{i}^{2+}$. As mentioned above, we can calculate these increments based on the total plasma current and build the equivalent geometry depicted in Figure 17. In this figure, the values of *v* are all representing velocity in the z-direction. If we subtract Equations (16) and (19) from each other and assume that (i) at the ionization point *i,* the z-velocity component of the primary beam and secondary beam are equal (it defines the secondary beam initial z-velocity) and (ii) that the path integral difference between the two consecutive secondary beams is negligible, i.e., the beams feel the same integral acceleration along their paths$,{\bar{a}}_{i}^{2+}$, then we can obtain the following algebraic relation:

(21) $$\;\;\;\;\;\;\;\;\;\;\;\;\;\;z_{d\left(i+1\right)}^{2+}-z_{d\left(i\right)}^{2+}=v_{\left(i+1\right)}^{+}T_{2\left(i+1\right)}-v_{\left(i\right)}^{+}T_{2\left(i\right)}+z_{\left(i+1\right)}^{+}-z_{\left(i\right)}^{+}$$

If we consider that the difference in the z-position of the two consecutive ionization points over the primary beam is much smaller than the difference in the z-position of the corresponding secondary ions on the detector, we can simplify the above equation to obtain

(22) $$∆z_{d}^{2+}=∆v^{+}{\bar{T}}_{2\;}$$

where ${\bar{T}}_{2\;}=\frac{1}{2}\left(T_{2\left(i+1\right)}+T_{2\left(i\right)}\right)$ is the average flight time of the two secondary beams. Dividing both sides by *dt,* which is the elemental time increment that the primary beam takes to travel between the two ionization points, we finally obtain

(23) $$\frac{∆z_{d}^{2+}}{{\bar{T}}_{2\;}.dt}=\frac{∆v^{+}}{dt}=a_{i}^{+}$$

This quantity is the primary beam z-acceleration taken at the location between the ionization points. Combining Equation (14) and the last equation above, we obtain the component of the magnetic poloidal field that is perpendicular to the primary beam velocity.

**Figure 18:**
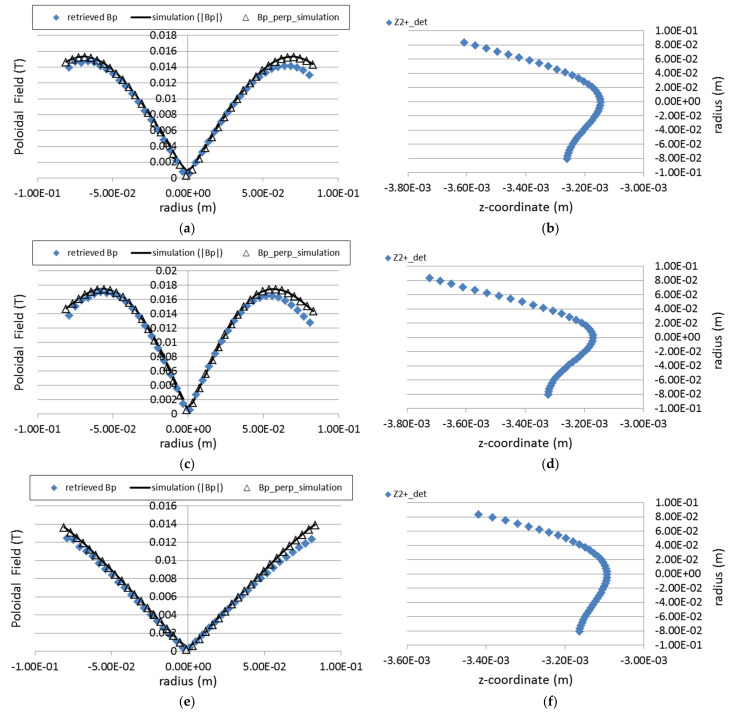
On the left side, figures show the retrieved values of the poloidal field profile (solid triangles) for three different current density profiles: (**a**) parabolic, (**c**) peaked (parabola square) and (**e**) flat. The values of the inputted profiles of the total poloidal filed and of the *B_p_* z-component are also displayed in the same graph. The figures on the right (**b**–**f**) show the corresponding detector z-positions of the secondary beams as a function of the radial location of the ionization volume.

We tested this iterative process for several symmetric current density profiles with a total plasma current of 6 kA. The results are summarized in Figure 18. As the poloidal field changes sign from the top half of the plasma to the bottom half, we plot the absolute values of *B_p_*. The proper sign can be recovered by considering the sense of the total plasma current. The method here presented can recover with great efficiency the absolute profile of the plasma magnetic poloidal field. An about 5–7% average of inaccuracy in absolute values can be observed for the radial range 2–2.5 cm of the plasma upper edge. This could be caused by the assumption that two secondary beams feel the same force while traveling over the plasma which may become less accurate when the travel times are longer.

In this evaluation of the method, we are not considering the detector spatial resolution. However, in practice, the finite size of the cells imposes an error of about 0.2–0.3 mm in the determination of the absolute z-position. This value already accounts for the beam profile distortion (that we can measure at the primary detector), the cell width (4 mm) and the noise on the detector cells.

For measuring fluctuations on the beams’ positions, as the beam profile remains unchangeable, the spatial accuracy can become about 0.1 mm, limited by noise that can be minimized by either using fast beam chopping or by subtracting the signal from neighboring cells (with similar noise content but without beam current). For instance, the magnetic field fluctuations induced by the toroidal field power supplies (50 Hz and 300 Hz) were characterized using the primary beam multiple-cell detector toroidal fluctuations.

This method was first proposed for ISTTOK in 1994 [14]. The first application to experimental results in ISTTOK was published in 1997 [15]. The method was later presented with more mathematic detail and used to estimate the effect of the finite size of the detector cells and beam profile in the retrieval of the poloidal field [16].

**Figure 19:**
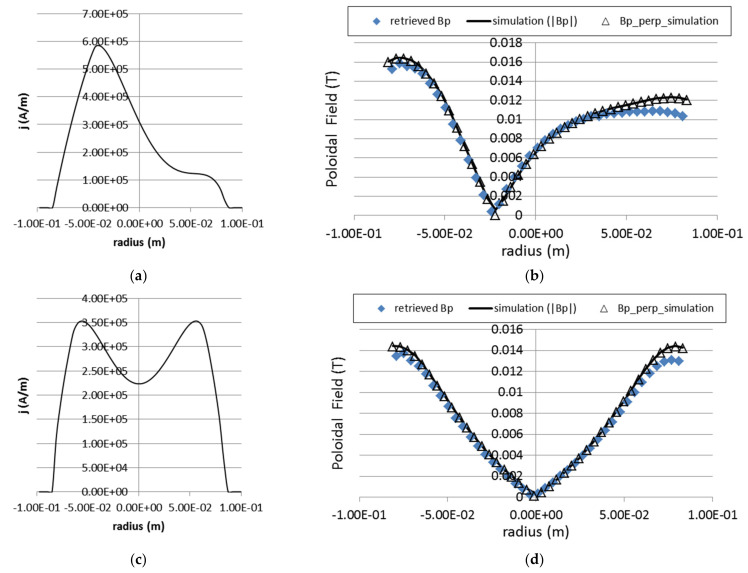
Retrieving of the poloidal field for an asymmetric and a hollow current density profiles. On the left graphs (**a**,**c**) are represented the inputted current density profiles and on the right (**b**,**d**) the retrieved values of the corresponding poloidal magnetic fields based only on the position of the secondary ions on the detector.

In spite of the initial assumptions for application to symmetric profiles, we tested the method for non-symmetric and for hollow current profiles. The results are depicted in Figure 19. They show that the method is still very effective in retrieving the corresponding poloidal field absolute values in such conditions.

**Figure 20:**
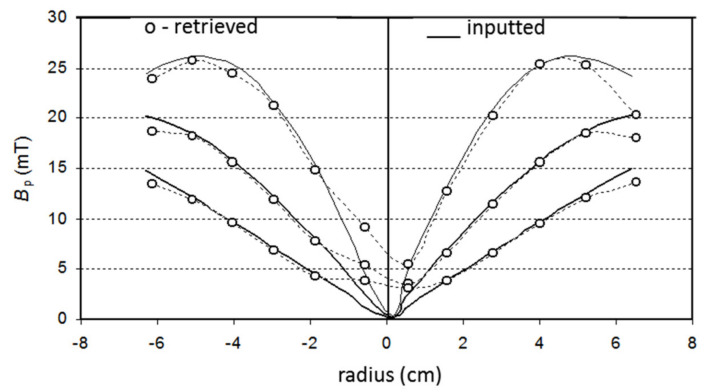
Retrieved poloidal field for three different poloidal field profiles (parabolic, parabolic squared and flat) taking into account beam profile distortion and finite cell size (12 × 5 MCAD with cell width of 4 mm).

In Figure 20 are depicted the retrieved poloidal field profiles for the same three plasma current density profiles as those used in Figure 18, considering the finite size of detector cells. The results show that even for the finite cell size, the retrieved values follow closely each of the poloidal field inputted profiles.

#### 3.3.2. Experimental Determination of the Evolution of the Plasma Current Magnetic Field Profile

**Figure 21:**
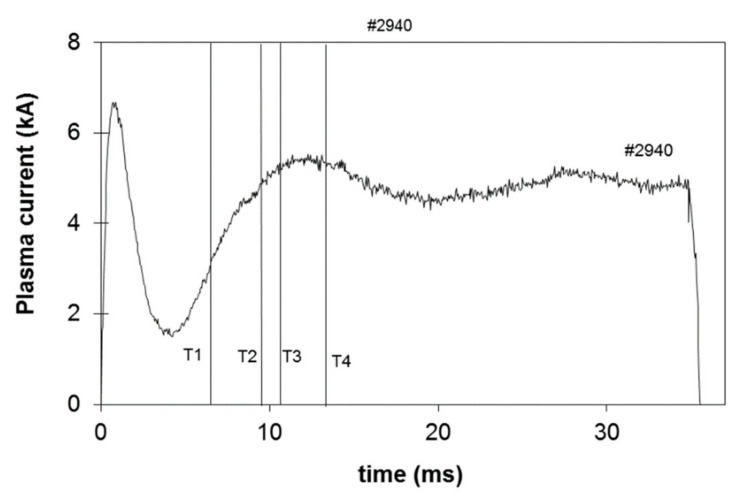
ISTTOK plasma current evolution and indication of the 4 instants when the poloidal field profile was determined (partially reprinted from [15]).

The evolution of the plasma current magnetic field profile was measured during ISTTOK discharge ramp-up [15]. In these particular experiments, the measurements are confined to the core region of the plasma due to the low signal on the more peripheral cells. A detector of 9 × 6 cells was used with a cell width of 2 mm. The measurements were taken at four time instances as indicated in the reference discharge in Figure 21.

**Figure 22:**
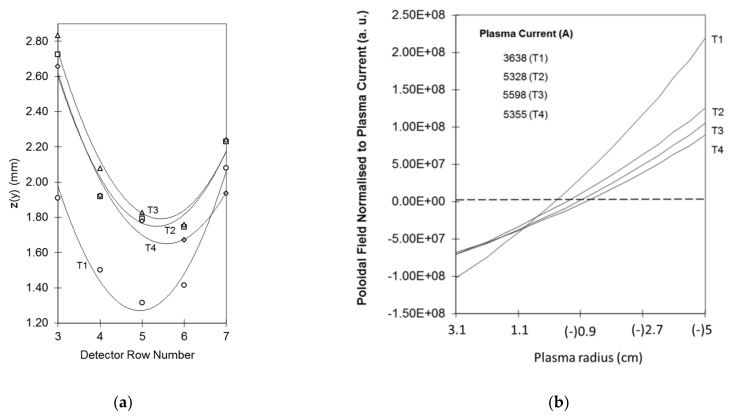
(**a**) The symbols indicate the measured z-positions of the secondary beams fitted with corresponding interpolation lines T1, T2, T3 and T4. (**b**) Estimated normalized poloidal field profiles from the detector characteristic lines at the time instantes T1, T2 T3 and T4 (reprinted from [15]).

The measured detector positions are depicted in Figure 22a. The positions are interpolated by a continuous function from which is computed the poloidal field profile, shown in Figure 22b.

The main observable result is that the plasma current increase is well followed by the secondary beam positions showing that the plasma is not centered in the earlier stages of the discharge. Near the current plateau, the plasma occupies the central position of the chamber, and the slightly decreasing slope of the poloidal field profile indicates that the current channel is becoming less peaked as the discharge evolves.

### 3.4. Plasma Potential Measurements

As was demonstrated above in Equation (2), the plasma potential affects the energy of the secondary beams. The Proca-Green 30° parallel plate energy analyzer has been used for measuring the secondary ion beam energy in the HIB-probe configuration. However, in order to keep the possibility to collect the whole cloud of secondary beams in the ISTTOK HIB-diagnostic configuration, it is necessary to use a different detector arrangement. To that end, the direction of the analyzing field in the energy analyzer must be parallel to the tokamak toroidal field. The most efficient way to save space and perform the required analyzing capability is to use a cylindrical energy analyzer. There are several experiments that require measuring ion beam energies where this type of analyzer is employed. In particular, it has been studied for application on the large helical device (LHD) for plasma potential measurements [17] (not implemented). However, the possible application of this analyzer in the LHD was limited to measuring one secondary beam at a time.

**Figure 23:**
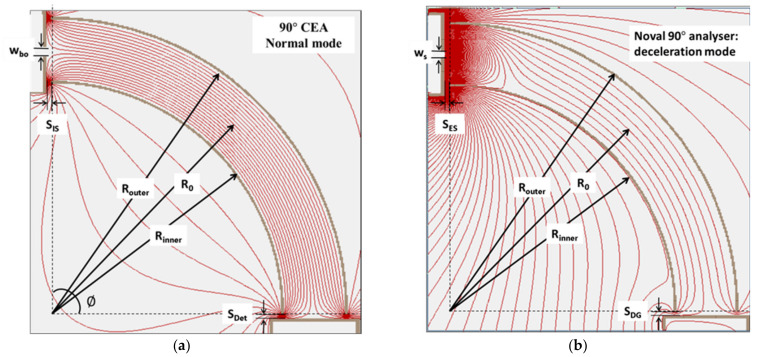
Representation of the equipotentials for the 90° cylindrical energy analyzer operating in normal mode (**a**) and the same analyzer operated in deceleration mode (**b**).

In ISTTOK, we adopted a compact 90° energy analyzer with the aim of collecting all secondary beams at once. Simulations showed that the beams could be collected from the whole plasma diameter. However, the attained energy resolution was relatively poor (in the range of several tens of volts) due to the short path length inside the analyzing field. To overcome this difficulty, a new mode of operation was proposed and successfully tested. This consisted in retarding the beam while it travels inside the analyzer [18] by applying a retarding field between the entrance (grounded) and at the back detector plane (positively biased mesh). In Figure 23, is presented the equipotential map of the normal and deceleration modes of operation.

In the normal operation, the radial electric field *E(R)* and the potential *ϕ(R)* inside the analyzer are described by

(24) $$E_{r}\left(R\right)=E_{0}\left(\frac{R_{0}}{R}\right)\;\varphi \left(R\right)=E_{0}R_{o}\ln\left(\frac{R}{R_{0}}\right){+}_{0}$$

where *E*_0_ and *ϕ*_0_ are the analyzer central values determined for the applied voltages to each electrode. For the normal mode operation, the required voltage applied between the electrodes in order to keep the beam in a specific trajectory is obtained by equalizing the electric and the centripetal forces

(25) $$∆V=2[\left(E_{0}-q\varphi_{0}\right)/q(\frac{d}{R_{0}})$$

If the analyzer is operated with symmetric plate voltages, the value of *ϕ*_o_ is zero. In the deceleration mode, the value of the central potential *ϕ*_o_ is strongly increased by offsetting the plates’ voltages. Consequently, the required electrode voltage difference to keep the beam in a particular trajectory is lowered in the deceleration mode as compared to normal mode operation. This is equivalent to obtaining a larger displacement on the beam orbit inside the analyzer in deceleration mode than in normal mode for a given change of beam energy if operating the analyzer at constant electrode voltages. In fact, the gain coefficient in beam displacement due to change in the beam energy *k*_E_ is given by (here we refer to the beam energy before it enters the analyzer)

(26) $$k_{E}=\frac{E_{beam}}{E_{beam}-q\varphi_{0\left(dec.\right)}}$$

For instance, for an input beam of 20 keV energy and a deceleration voltage of 8 kV, we obtain a coefficient gain of *k*_E_ = 5 (*q* = 2). This translates into an increased beam displacement in the detector of five times more than in normal mode. Other factors concur to limit this displacement gain such as beam profile stretching and the internal asymmetry of the electric field while the beam changes orbit. Taking all these factors into account, a magnification of M = 4 was obtained in the sensitivity to beam energy changes as compared to the normal case. A demonstration of this principle was performed using an electron beam, and the results matched the simulations [19].

**Figure 24:**
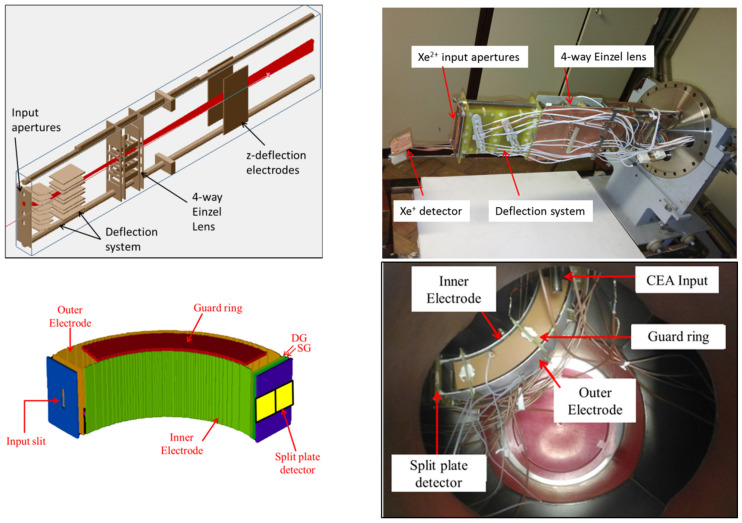
Components of the detection system: *Xe^2+^* deflection system with Einzel lens (**top row**) and cylindrical energy analyzer (**bottom row**).

In spite of it being possible to collect all secondary beams with this geometry, the practical placement of the analyzer was only possible away from the exit port of the tokamak. This implied the need to deflect the secondary beams from the port exit to the cylindrical energy analyzer entrance. The measurements are taken from four sample volumes in the plasma to fit in the available space in the deflection system. The full geometry is illustrated in Figure 24.

**Figure 25:**
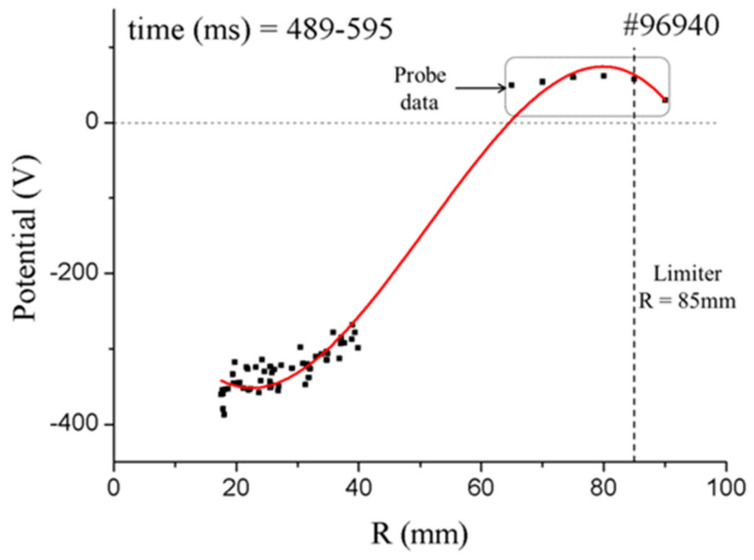
Plasma potential measurement by the HIBD in the plasma core of ISTTOK (reprinted from [20]).

Experimental results (Figure 25) in the measurements of plasma potential in the core region are reported in [20]. In these test experiments, only the central channel was used due to limitations on the number of power supplies and amplifiers. The plasma was scanned by means of the vertical field coils, and a spatial range of values for the plasma potential was obtained. The plasma potential absolute values were calibrated with an indirect method implying up to ±70 V in absolute plasma potential error. As for the relative error, experiments demonstrate ±25 V or, equivalently, an upper limit of *ΔE*/*E* ~ 2 × 10^−3^ for the CEA sensitivity to the plasma fluctuation measurements.

### 3.5. Simultaneous Measurements of Pressure-like, B_p_ and V_p_ Fluctuations

The most recent developments on the ISTTOK HIBD were pursued aiming at achieving the possibility to measure simultaneously the profiles of plasma pressure-like (*n sigma)*, plasma current magnetic field and plasma potential. This capability was tested by making the simultaneous measurements of the fluctuations of these three parameters via the fluctuations of the beam signals.

**Figure 26:**
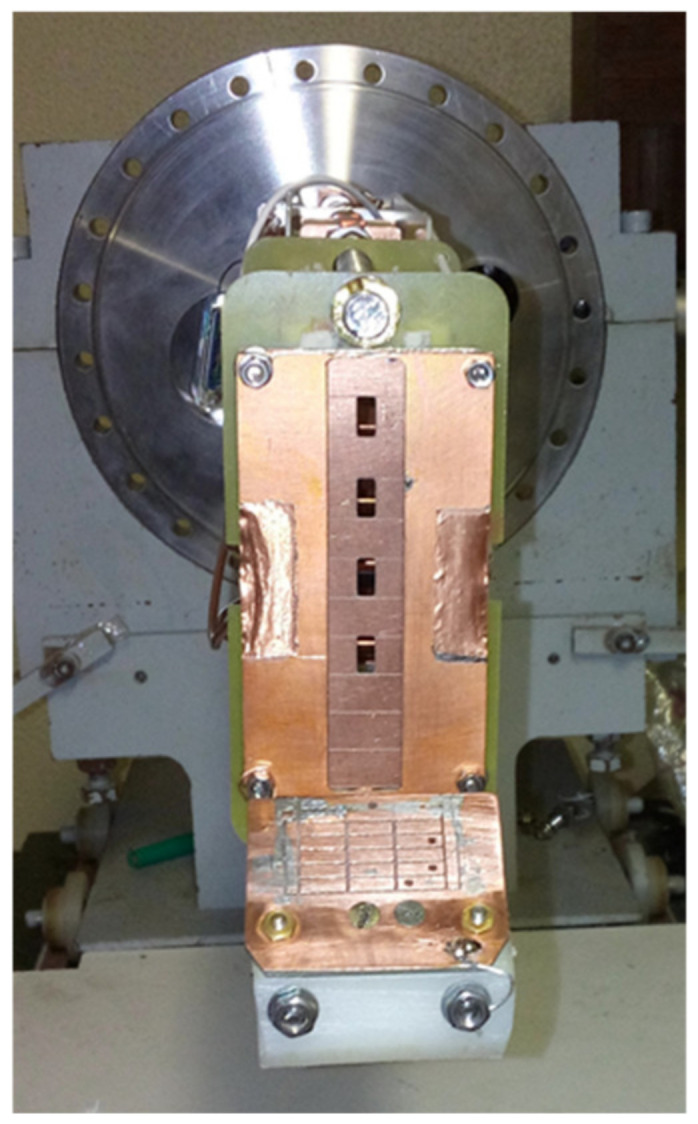
Front MCAD for measuring the X^2+^ z-positions. Four apertures in the front MCAD allow for the passage of four secondary beams that are directed to the cylindrical energy analyzer, placed behind the circular flange shown in the picture.

The detector for measuring the z-displacements of the secondary beams was made of 12 rows by 3 column cells, each with 4 mm width. This detector is placed at the exit port of the tokamak beyond the deflection electrode unit. Some of the central column cells were removed to let some secondary ions pass into the CEA (Figure 26). Signals were acquired in the CEA through the central opened channel number 3, counting from the top. The position of the secondary ions was measured by the balance of currents in the left and right cells in the front detector weighted by the total current collected at the front detector plus the current of the same beam collected at the back detector, behind the CEA exit. Due to the intrinsic deceleration operation mode properties, any changes in input angle in the z-direction at the CEA entrance are not transferred to a change in the z-position of the beam at the analyzer back detector [18] (angular first-order focus). These results allow us to separate the z-position displacements due to poloidal field changes at the front detector from those measured at the back of the CEA, which will be due to the changes in beam energy, i.e., plasma potential.

**Figure 27:**
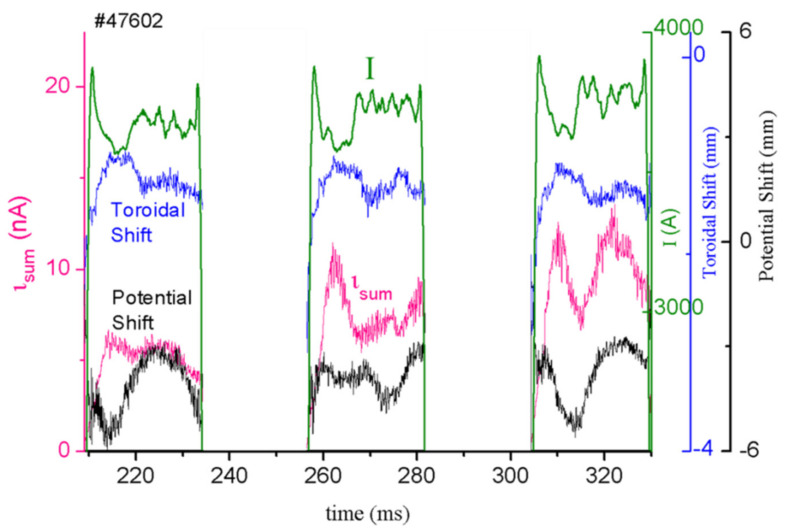
Raw signals of the fluctuations of the secondary beam position and intensity at the CEA front and back detectors.

The raw results on the beam signals for three positive cycles of an AC discharge are presented in Figure 27. The data show the z-displacements of the secondary beam due to plasma potential variations (potential shift) and plasma poloidal field (toroidal shift) simultaneous with the pressure-like fluctuations (current fluctuations of the secondary beam). Cross-correlation analyses between the z-displacements measured at the front (plasma-current-driven) and back (plasma-potential-driven) detectors showed no correlation in the fluctuation of these signals, indicating that these two measurements can be performed independently.

## 4. Conclusions and Future Work

The ISTTOK HIBD is based on a unique configuration that allows the collection in a multiple-cell array detector of the probing secondary beams generated from the whole plasma diameter. The wealth of information obtained allows accounting for the path integral effects and retrieving the local values of the plasma parameters at the ionization volume in the plasma. The method allowed obtaining the profiles of plasma-like pressure with good spectral resolution. Exploitation of the plasma pressure-like measurements in AC discharges allowed for the identification and characterization of MHD activity and turbulent transport in edge polarization experiments. Further, the individual profiles of temperature and plasma density were obtained utilizing a *Xe^+^* and *Hg^+^* beam combination.

The real-time determination of the plasma column center using the distribution of the measured ‘*n sigma’* profile was successfully used to control the vertical position of the plasma column.

An innovative method to determine the plasma current poloidal magnetic field was here presented and tested. The accuracy of the method allows identifying different profiles of plasma current density. The method was successfully applied in the identification of the poloidal field profile evolution during the ramp-up phase of the plasma discharge.

The most recent developments addressed the measurements of the plasma potential using a cylindrical energy analyzer. The main difference from other experiments using this device is the introduction of the deceleration mode of operation. The predicted gains in the beam energy sensitivity can be tuned with the biasing voltage to reach nearly five times better resolutions. In principle, this analyzer configuration allows for measurements from several points along the primary beam path, inside the plasma. Experimental results for the core measurements were obtained with a relative precision of ±25 V, whereas indirect calibration allowed an absolute with errors of ±70 V. The preliminary results for the simultaneous measurements of the plasma potential, poloidal magnetic field and pressure-like fluctuations indicate that these measurements can be made without crosstalk. They pave the way for extending the coverage to more points in the plasma to allow for performing transport and turbulence analysis along the primary beam path with preservation of the phase.

The future work foresees the installation of additional amplifiers and power supplies in order to use the full capabilities of the CEA and cover more sample volumes in the plasma. When in full operation, it would be used to confront the experimental measurements of kinetic and magnetic pressure fluctuations with the theoretical and numerical predictions. By keeping the phase information, it adds a new ingredient which would be instrumental for providing time-correlated information on the interplay between turbulence, particle transport and MHD activity.

## Data Availability

All data used is indicated in the references given or is explicitly included in the paper as the only main source. No other public sources of data exist.
